# Supplementary material for: SyNDI: synchronous network data integration framework
Source: BMC Bioinformatics. 2018 Nov 6;19:403. doi: 10.1186/s12859-018-2426-5 (PMC6219086; doi:10.1186/s12859-018-2426-5)
Supplement: Supplementary file 5 — Files of the example presented in Additional file 4. This additional file is a zip package that contains all files used in Additional file 4. Detailed descriptions of each of these files are presented in a README file included in this zip package. (GZ 30411 kb) [file 12859_2018_2426_MOESM5_ESM.gz › SyNDITools/Meme2Fimo/meme_4.9.0_4.tar.gz!/meme_4.9.0/website/html/mhmm-faq.in]

Meta-MEME v3.3


> ### Frequently asked questions
>
> ---
>
> - **Why can't the Meta-MEME program 'mhmm' read my MEME output
>   file?**
>
>   Meta-MEME can only read the HTML version of MEME ouput.
>   Also, Meta-MEME cannot read MEME files that contain both
>   positive and negative motifs. If you don't know what this means,
>   don't worry about it. Only advanced MEME users are likely to
>   produce files containing positive and negative motifs. In the
>   future, Meta-MEME will be capable of processing these motifs.
> - **Why doesn't Meta-MEME include in the hidden Markov model all
>   of the motifs that I requested?**
>
>   This is generally only a problem if you have downloaded the
>   Meta-MEME source code and installed it locally.
>
>   When building a linear HMM, Meta-MEME uses output from MEME to
>   set the order and spacing of motifs within the model. Meta-MEME
>   therefore requires that the MEME output provided to 'mhmm' contains
>   at least one motif occurence diagram that contains exactly one
>   occurence of each of the selected motifs. Occurences of motifs that
>   were not requested are ignored.
>
>   If, for example, you request that the model contain motifs 1, 2
>   and 3, but the MEME output file contains only these diagrams
>
>   ```
>      30-[1]-23-[3]-1
>      28-[1]-32
>      40-[2]-2-[3]-1
>      2-[1]-[1]-4-[2]-[3]-3
>   ```
>
>   then 'mhmm' will be unable to find a representative diagram. The
>   program will then throw out the last requested motif and re-search
>   the diagrams, repeating this procedure until it finds a diagram
>   that works. In this case, there is no diagram containing motifs 1
>   and 2 either, so the final model would be based upon the first
>   diagram and would contain only motif 1.
> - **All of the scores from my Meta-MEME search are below zero.
>   What does this mean, and how do I fix it?**
>
>   Occasionally, Meta-MEME has a tendency to improperly scale the
>   log-odds scores that it reports. This is a result of the mismatch
>   between the topology of the foreground and background models. The
>   problem is especially likely when Viterbi, rather than total
>   probability, log-odds scores are computed. You can try computing
>   total probability scores instead, or turn off the training portion
>   of Meta-MEME to create a model that is less finely-tuned to your
>   particular training set.
>
> ---
>
> Return to the Meta-MEME home page.
>
> *Please send comments and questions to:
> @METAMEME\_CONTACT@.*
